# Crop pathogen emergence and evolution in agro-ecological landscapes

**DOI:** 10.1111/eva.12251

**Published:** 2015-03-05

**Authors:** Julien Papaïx, Jeremy J Burdon, Jiasui Zhan, Peter H Thrall

**Affiliations:** 1 UMR 1290 BIOGER, INRAThiverval-Grignon, France; 2 UR 341 MIA, INRAJouy-en-Josas, France; 3 UR 546 BioSP, INRAAvignon, France; 4 CSIRO Agriculture FlagshipCanberra, ACT, Australia; 5 Fujian Key Lab of Plant Virology, Institute of Plant Virology, Fujian Agriculture and Forestry UniversityFuzhou, China

**Keywords:** agro-ecological interface, landscape epidemiology, pathogen evolution, plant disease management

## Abstract

Remnant areas hosting natural vegetation in agricultural landscapes can impact the disease epidemiology and evolutionary dynamics of crop pathogens. However, the potential consequences for crop diseases of the composition, the spatial configuration and the persistence time of the agro-ecological interface – the area where crops and remnant vegetation are in contact – have been poorly studied. Here, we develop a demographic–genetic simulation model to study how the spatial and temporal distribution of remnant wild vegetation patches embedded in an agricultural landscape can drive the emergence of a crop pathogen and its subsequent specialization on the crop host. We found that landscape structures that promoted larger pathogen populations on the wild host facilitated the emergence of a crop pathogen, but such landscape structures also reduced the potential for the pathogen population to adapt to the crop. In addition, the evolutionary trajectory of the pathogen population was determined by interactions between the factors describing the landscape structure and those describing the pathogen life histories. Our study contributes to a better understanding of how the shift of land-use patterns in agricultural landscapes might influence crop diseases to provide predictive tools to evaluate management practices.

## Introduction

Integrating ecosystem processes occurring at large spatial scales into the design of agricultural landscapes with the aim of improving productivity while decreasing the negative impact of agricultural practices on the environment is increasingly recognized as key to addressing global food security concerns (Bianchi et al. 2006; Tscharntke et al. 2012; Bommarco et al. 2013). At the landscape scale, remnant areas hosting wild vegetation (weeds, exotic or native plant communities) have the potential to promote desired ecosystem services because of their influence on the community ecology of crop pests and beneficial organisms such as pollinators and predators (Bianchi et al. 2006; Chaplin-Kramer et al. 2011), and through their impact on the disease epidemiology and evolutionary dynamics of crop pathogens (Wisler and Norris 2005; Burdon and Thrall 2008; Alexander et al. 2014). However, management plans to hinder the evolution of crop pests are rarely designed at the landscape scale and if so, they do not consider remnant patches of wild vegetation. A classic example in plant–pathogen interactions was the campaign to eradicate barberry (*Berberis vulgaris*) growing along wheat field margins in the United States (Roelfs 1982; Kolmer et al. 2007). Barberry is the sexual host for wheat stem rust (*Puccinia graminis tritici*) and when present, provides early inoculum and new infectivity combinations for the pathogen. In this study, we quantified the impacts of the area, spatial configuration and average persistence time of wild vegetation fragments in agricultural landscapes on the emergence of crop pathogen over time.

In plant epidemiology, the breakdown of qualitative resistance in crop cultivars can lead to spectacular disease outbreaks (McDonald and Linde 2002) and the erosion of quantitative resistance causes partly resistant hosts to become increasingly susceptible (Lannou 2012). The role of wild hosts in influencing the outcome of such evolutionary processes has been reported for various plant–pathogen systems with different possible scenarios (Jones 2009; Alexander et al. 2014) including: emergence of pathogens from native flora (Wang et al. 2010; van der Merwe et al. 2013), use of sources of resistance from wild hosts (Garry et al. 2005; Lebeda et al. 2008; Leroy et al. 2014) and reciprocal influence of native flora and cultivated plants (Webster et al. 2007; Lê Van et al. 2011). The importance of considering interactions across the wild and cultivated compartments in agricultural landscapes is clearly seen in the emergence of Fusarium wilt disease in Australian cotton-growing regions in the early 1990s (*Fusarium oxysporum* f. sp. *vasinfectum* on *Gossypium hirsutum* L.). Several *Gossypium* species are native to Australia, and at least two of these species have distributions that overlap cultivated cotton-growing regions. Isolates of *F. oxysporum* from these wild hosts were found to cause mild symptoms of Fusarium wilt in cultivated cotton (Wang et al. 2004). A detailed study of the genetic structure of *Fusarium oxysporum* f. sp. *vasinfectum* and *F. oxysporum* populations indicated that Fusarium wilt in cultivated cotton evolved locally through specialization of native *F. oxysporum* strains to the crop (Wang et al. 2010).

**Figure 1:**
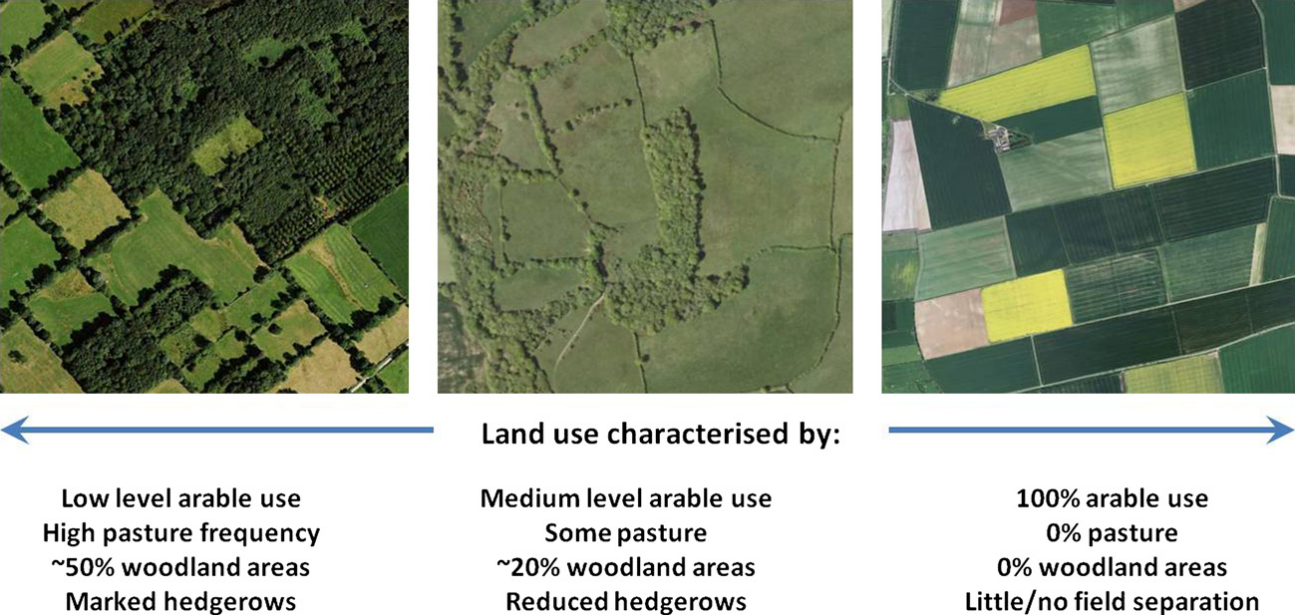
Spatial structure of the agro-ecological interface across different farming systems (Imagery ©2014 Cnes/Spot Image, Digital Globe; Map data ©2014 Google).

Changes in agricultural practices imply changes in the potential for contact between crops and remnant vegetation in agricultural landscapes. The area where such contacts occur is termed the agro-ecological (AE) interface (Burdon and Thrall 2008). Variation in AE interfaces among farming systems implies highly diverse situations for crops and wild plants to interact (Fig.1). Wild and cultivated elements can be either almost undistinguishable and highly intricately intermeshed such as in tropical agroforestry landscapes (Tscharntke et al. 2011) or strongly separated with markedly different species diversity such as in intensive monoculture landscapes. The area and spatial distribution of remnant wildlands can vary greatly: for example, in some European regions, landscapes are composed of small fields separated by hedges with significant areas of woodland and the AE interface is highly developed. In contrast, this interface is extremely reduced in agricultural regions dominated by intensive monoculture of broadacre crops (Fig.1). Seasonality can also differ between the agricultural and wild elements of these landscapes, with grasses and herbs in hedgerows providing year-long green bridges when annual crop cycles finish and fields are fallowed.

Variation in the temporal (through seasonal changes) and spatial (through landscape composition and organization) structure of agro-ecological systems and the extent and complexity of the AE interface can have direct consequences for pathogen eco-evolutionary dynamics. Seasonal fluctuations in environmental conditions can affect pathogen spread and persistence (Altizer et al. 2006). In particular, periodic host absence in agricultural landscapes forces plant pathogens to survive on volunteer plants (wild relatives, alternative hosts, seedlings) or to have specific life-history strategies (saprophyte, free-living stages) that may lead to drastic reductions in pathogen population size [see Suffert et al. (2011) for an example on *Septoria tritici* blotch]. In addition, such pulses in host density have important consequences for pathogen evolution (van den Berg et al. 2011; Hamelin et al. 2011; Zhan et al. 2014). On the other hand, the diversity and spatial arrangement of host genotypes have been shown to influence disease spread and persistence at the field scale in variety mixtures (Mundt and Leonard 1986; Skelsey et al. 2005; Mundt et al. 2011) and to extend its influence at the landscape scale by modifying pathogen habitat connectivity (Zhu et al. 2000; Skelsey et al. 2010; Papaïx et al. 2014c). Pathogen evolution is also sensitive to the spatial heterogeneity of agricultural landscapes (Zhan et al. 2002; Stukenbrock and McDonald 2008; Sommerhalder et al. 2011) with large uniform areas facilitating the evolution of pathogen specialization and genotypes that are better adapted to crop hosts (Débarre and Gandon 2010; Papaïx et al. 2013).

Despite the broad range of work published on pathogen evolution in agricultural landscapes (Stukenbrock and McDonald 2008; Burdon et al. 2014), the potential consequences of spatio-temporal variation in the structure of the agro-ecological interface for the eco-evolutionary dynamics of plant diseases have been poorly understood (Burdon and Thrall 2008; Alexander et al. 2014). Indeed, most previous studies did not consider the role of remnant wild vegetation but focused on the cultivated crop. To our knowledge, only one modelling study considers both wild and cultivated hosts but do not take into account the explicit spatial configuration of the landscape (Fabre et al. 2012). The study involved a gene-for-gene system for virus epidemics in a landscape composed of a susceptible cultivar, a resistant cultivar and a wild reservoir. The reservoir was assumed to be selectively neutral and allowed the pathogen to survive during the off season. They found that epidemic intensity was the main factor explaining resistance breakdown. The landscape composition (cropping ratio between the susceptible and resistant cultivars) was also found to be crucial but even its influence was determined by epidemic intensity. However, the assumption that the wild reservoir was selectively neutral and the presence of a susceptible crop that increases pathogen population size prevented Fabre et al. (2012) highlighting any role resulting from viral dynamics in the reservoir.

Finding novel resistance genes and integrating them into new crop varieties is a long and costly process. Hence, the development of effective strategies for the deployment of resistant genotypes that increase resistance gene durability should be a central goal of epidemiological studies of crop host–pathogen interactions (Burdon et al. 2014). Developing such strategies requires that we better understand how different agro-ecological landscapes (considering both wild and cultivated elements) might influence the spread and evolution of crop diseases in order to provide predictive tools to evaluate management practices (Shennan 2008; Thrall et al. 2011). Indeed, the shift of land-use patterns in agricultural landscapes through intensification or extensification of agricultural production systems modifies the interface between the agro-ecological elements, and we need to understand the potential consequences of such modifications for a better management of landscapes and control of crop pathogens.

Here, we develop a demographic–genetic simulation model to study how the spatial and temporal distribution of remnant wild vegetation patches embedded in an agricultural landscape can drive the emergence of a crop pathogen and its subsequent specialization on the crop host. In this study, we take a first step towards modelling more complex situations by considering a relatively simple system where the crop and the wild plant are each represented by only one genotype. We first present the model and the simulation experiment. Then, we study the evolutionary trajectory of the pathogen population in different agro-ecological landscapes. We characterized the landscape structures (spatial configurations and duration of the cropping season) that favoured crop pathogen emergence and studied how different life-history traits (dispersal ability and trade-off in aggressiveness) of the pathogen could affect these outcomes. Finally, we discuss the implications for the management of remnant elements in agricultural landscapes.

## Model and methods

### Model overview

Our model describes the numerical dynamics and the evolutionary changes in the genetic composition of a pathogen population thriving on two different hosts: a wild host and a crop. The wild host inhabits patches of remnant vegetation embedded in an agricultural landscape composed of paddocks where the crop is sown. The approach we used allows us to control the spatial aggregation of wild patches and the area they covered. The wild host disperses among the patches of remnant vegetation and is present all year round. In contrast, the crop grows locally and is present only during the cropping season. As a consequence, the pathogen depends upon the wild host to bridge the off season, when the crop has been harvested. The pathogen disperses passively across the whole landscape (e.g. through wind dispersed propagules) regardless of the host type from which it is dispersed or the one on which it lands.

Pathogen genotypes are characterized by their aggressiveness on the host types. Aggressiveness is used here to describe the quantitative interaction between a pathogen genotype and a host type reflecting, for example, differences in spore infection efficacy, lesion development rate, time from infection to sporulation and the abundance of spores produced. Thus, aggressiveness is a composite trait directly linked to the fitness of a pathogen genotype on a given host type and to the potential amount of disease the host suffers in the presence of that pathogen genotype. Importantly, we consider a trade-off in aggressiveness on the two host types. Such trade-offs would reflect the constraint for the pathogen of simultaneously investing in different traits: the allocation of limited resources in one trait has a negative impact on another trait (Pariaud et al. 2009; García-Arenal and Fraile 2013; Laine and Barrès 2013). At the beginning of a simulation, the pathogen population is only adapted to the wild host and cannot attack the crop. However, new pathogen genotypes can arise through mutation resulting first in the emergence of genotypes able to attack the crop and then in a gradual increase in aggressiveness on the crop.

**Figure 2:**
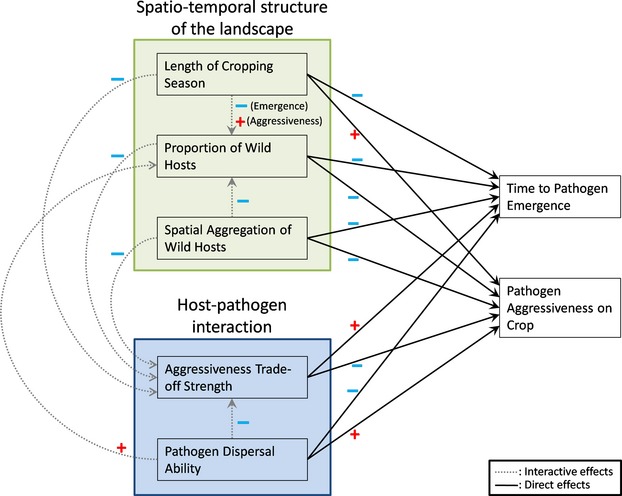
Summary of the direct (black solid arrows) and interactive (grey dashed arrows) effects of the input factors on the variables describing the pathogen evolution.

Using this modelling framework, we quantify the impacts of the area and spatial configuration of the wild remnant patches as well as the duration of the cropping season on the emergence of crop pathogen and its subsequent specialization on the cultivated host over time (Fig.2). We also investigate how the life history of the pathogen (i.e. strength of the trade-off in aggressiveness and dispersal ability) mediates the effect of spatial and temporal habitat variability on crop pathogen emergence (Fig.2).

### Population dynamics and pathogen evolution

#### Spatial structure

**Figure 3:**
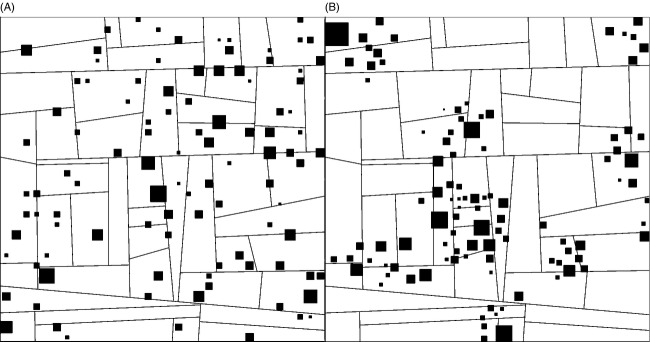
Examples of simulated landscapes composed of 49 paddocks (solid lines) and 100 wild patches (black squares). The wild host population covers 5% of the total landscape surface, and wild patches are randomly distributed (A) or clustered (B) in space.

The spatial structure of the system within which hosts and pathogens interact is represented as a two-dimensional metapopulation composed of patches of two types: cultivated and wild (Fig.3). The plant hosts are represented by two species: the crop is present in cultivated fields and the wild plant in wild remnant patches. Cultivated areas are generated by simulating a set of crop paddocks using a T-tessellation algorithm that makes it possible to control the size, number and shape of paddocks. This algorithm is based on the Metropolis–Hastings–Green principle that makes it possible to generate several landscape replicates sharing the same characteristics (Kiêu et al. 2013; Papaïx et al. 2014a). Wild patches are then positioned on that agricultural landscape with spatial aggregation (random and clustered – Fig.3A,B) and the total surface covered (2.5%, 5% and 10%) determined as model inputs (Table1). Each wild patch centre was first located, and then surfaces were drawn from a log-normal distribution to obtain the desired percentage of landscape coverage by wild vegetation. For each combination of wild patch aggregation and surface coverage values, five different landscapes composed of 100 wild patches and, respectively, 52, 49, 51, 48 and 52 paddocks were constructed and used as landscape replicates.

**Table 1:** Definitions of the main terms and parameters used in modelling

Symbols	Description	Value
(1) Spatial and temporal structure		
−	Number of paddocks	52, 49, 51, 48 and 52
−	Number of wild patches	100
*q*	Proportion of the agricultural landscape covered by the wild metapopulation	2.5%, 5% and 10%
*N*	Number of years	50
*Y*	Number of days in a year	360
*T*	Number of days in a cropping season	Between 60 and 300
(2) Crop and wild host dynamics		
*δ*^*c*^	Crop growth rate	0.1
*K*^*c*^	Carrying capacity of cultivated patches	Proportional to the paddock surface
*S*_0,*c*_	Number of susceptible plants initiating the cropping season	10% of *K*^*c*^
*r*^*w*^	Wild host reproduction rate	1 by susceptible plant by day
*K*^*w*^	Carrying capacity of wild patches	Proportional to the wild patch surface
*d*^*w*^	Wild host death rate	0.1
*distM*^*w*^	Wild host mean dispersal distance	10% of the landscape scale
(3) Pathogen population and evolutionary dynamics		
*β*	Shape of the trade-off function	0.6, 1 and 1.4
*e*	Pathogen infection efficiency	Varying according to the trade-off function. The maximal value for *e* is 0.4.
*r*^*P*^	Pathogen reproduction rate	2 by infected plant by day
*d*^I^	Infected plant death rate	0.1
*m*	Pathogen mutation rate	0.002 towards the 2 adjacent genotypes and 0.004 for the fully specialized genotypes
*distM*^P^	Pathogen mean dispersal distance	2.5%, 10% and 25% of the landscape scale

#### Temporal structure

**Figure 4:**
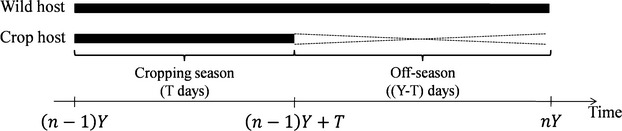
Schematic representation of the model temporal structure for one year. While the wild host is present all year round, the crop is cultivated only during the cropping season. *n*, index of the current year; *Y*, number of days during a year; *T*, cropping season duration.

The epidemiological model we used is characterized by a temporal cycle (i.e. a year) composed of two time periods: the cropping season and the off season (Fig.4). During the cropping season both the crop and wild hosts are present in the landscape. Conversely, during the off season, only the wild host is present. The seasons are separated by discrete events such as crop harvest or planting. Epidemics were simulated over *N* = 50 years composed of *Y* = 360 time steps (i.e. days). The cropping season forms the first *T* days of the year, whereas the off season is represented by the remaining *Y* − *T* days of the year. The cropping season duration, *T*, was varied from 60 to 300 days at 60-day intervals. We assumed here that, within a given simulation run, the spatial structure remains fixed across years.

#### Eco-evolutionary dynamics

We present here a semi-discrete (Mailleret and Lemesle 2009) and deterministic version of the susceptible-infectious (SI) model used in the simulation experiment, which describes the dynamics of the densities of susceptible (*S*) and infectious (*I*) host plants in each local patch within the metapopulation. Let the subscripts *i* and *n* indicate the patch and year respectively, the subscripts *c* and *w* the crop and wild host species, respectively, and the subscript *p* the pathogen genotype. Let 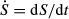, and *t*^−^ and *t*^+^ denote the time intervals immediately before and after time *t*, respectively. We also define *C* and *W* as the set of cultivated and wild patches, respectively. Table1 summarizes the main terms and parameters used.

During the cropping season, both hosts are present and the dynamics for the cultivated patches are as follows:

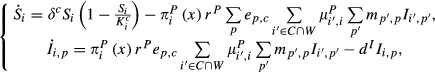
1 for all *t* between (*n*–1)*Y*, and (*n*−1)*Y* + *T*, *n* = 1, 2, …, for all *i* ε *C*. *δ*^c^ is the growth rate of the crop, 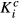 is the carrying capacity of patch *i* and is proportional to the area of that patch, *r*^*P*^ is the number of pathogen propagules produced by one infected plant per day, *e*_*p,c*_ is the infection efficiency of pathogen genotype *p* for the crop, 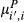 is the pathogen dispersal rate from patch *i*′ to patch *i*, 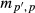 is the pathogen mutation rate from genotype *p*′ to genotype *p*, and *d*^I^ is the death rate of an infected plant. 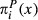 is the proportion of pathogen propagules that come into contact with a susceptible plant. This is an increasing function of 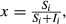 the proportion of susceptible plants in the patch. We consider the following sigmoid function for 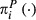:

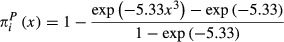
3 giving an inflection point for *x* ≈ 0.5. The function 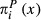 ensures that, in patch *i*, 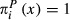 if all the plants are susceptible and 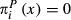 if there are no susceptible plants. In contrast to the crop, the wild host can disperse among remnant patches, which leads to the following dynamics for the host–pathogen interaction in wild patches:

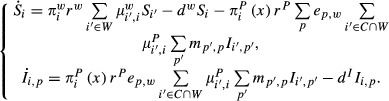
4 for all *i*∈*W*. The rate of plant propagule establishment is represented by 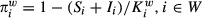, *i* ∈ *W*, where 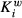 is the carrying capacity of patch *i*. It is equal to 0 if there is no available space (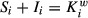), and to 1 if the patch is unoccupied. *r*^*w*^ is the number of plant propagules produced by one susceptible plant per day, 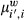 is the wild host dispersal rate from patch *i*′ to patch *i*, *d*^*w*^ is the death rate of susceptible plants, and *e*_*p*,*w*_ is the infection efficiency of pathogen genotype *p* for the wild host.

The transition between the cropping and the off season was carried out by removing all of the crop in cultivated areas, but keeping the state of the wild patches unchanged. During the off season, only the wild host is present and its dynamics are the same as during the cropping season (eqn 4 – for all *t* between 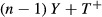 and *nY*). Finally, the transition between the off- and the new cropping season is described by:

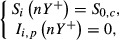
5 for all *i* ∈ *C*. *S*_0,*c*_ is the number of susceptible plants of the crop initiating the cropping season. The state of the wild patches is kept unchanged.

#### Dispersal

While the crop can only grow locally (i.e. where it has been sown) the wild plant and the pathogen can both disperse. Dispersal rates among patches are computed from an individual dispersal function. The propagule density emitted from a given source point *z* and arriving at a given reception point z′ is given by:

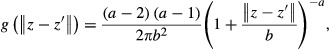
6 where 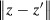 is the Euclidean distance between *z* and *z*′, *b* > 0 is a scale parameter and *a* > 2 determine the length of the dispersal tail: the lower the value of *a*, the longer the dispersal tail, and the more probable long distance dispersal events are. The mean dispersal distance travelled by a propagule is defined only when *a* > 3 as *distM* = 2*b*/(*a*−3). The expression of *distM* made it possible to vary the mean dispersal distance while keeping the probability of long distance dispersal events (parameter *a*) fixed. We thus considered three values for the mean dispersal distance of the pathogen (*distM*^*P*^ = 2.5%, *distM*^*P*^ = 10% and *distM*^*P*^ = 25% of the landscape scale) by varying the scale parameter *b* and fixing *a* at 3.4. The mean dispersal distance of the wild host was fixed at *distM*^*w*^ = 10% of the landscape scale.

From eqn 6, the probability of a propagule dispersing from patch *i* to patch *j* is computed by performing the integration of the dispersal function *g*(·) between pairs of points that belong to the areas *A*_*i*_ and *A*_*j*_ of patches *i* and *j*, respectively (Bouvier et al. 2009):

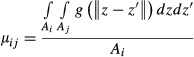
7

In eqn 7, the integral of *g*(·) is divided by the area of the originating patch to ensure 0 ≤ *μ*_*ij*_* ≤ 1*.

#### Pathogen population genetic structure

Pathogen genotypes are characterized by their aggressiveness on each host species. The aggressiveness of pathogen genotype *p* on host species *h* is defined by its nonspatial basic reproductive number in a monomorphic host population of plant species *h*, 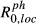. In epidemiology, the basic reproductive number is a classical measure of pathogen fitness. It represents the number of secondary infections arising from a single infected individual in a fully susceptible host population and thus reflects the potential disease severity the pathogen can cause to its host. Note that the pathogen population goes extinct if its basic reproductive number is below unity. In our system, the aggressiveness of pathogen genotype *p* on host species *h* is thus defined by 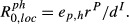


We assume a trade-off in pathogen aggressiveness on the two host species: a gain in pathogen aggressiveness on the crop has a cost in terms of reduced aggressiveness on the wild host (and vice-versa). Thus for a generalist pathogen strain, performance on any particular host species is less than that achieved by the pathogen genotype specifically specialized to that host but is greater than the performance of the pathogen genotype specialized on the other host species. Gain and cost are linked through the relationship:

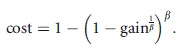
8

The parameter *β* determines the global concavity of the trade-off curve: the curve is concave when *β* is below unity, linear when *β* = 1 and convex otherwise. We will refer hereafter to concave curves as weak trade-offs, because they correspond to cases where the cost of being a generalist is low. Similarly, convex curves will be called strong trade-offs (Ravigné et al. 2009). In the simulation experiment, we fixed *r*^*P*^ = 2, *d*^*I*^ = 0.1 and the maximal infection efficiency at 0.4. The infection efficiencies of the other pathogen genotypes were computed from eqn 8, by varying the gain in aggressiveness between 0% and 100% and by considering three values for the trade-off shape, *β* = 0.6, *β* = 1 and *β* = 1.4.

The pathogen population is initially composed of one genotype but other pathogen genotypes can arise through mutation. We assume that the pathogen population evolves gradually: a new genotype arises from closely related genotypes by mutation with small gains or losses in *R*_0,*loc*_. The probability that a pathogen propagule was of the same genotype as its parental individual was set to *m*_*pp*_ = 0.996, and then, we set 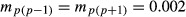. Exceptions were the pathogen genotypes with the highest aggressiveness on either the crop or the wild host – these mutated towards less specialized genotypes with a probability of 0.004 to keep their overall mutation rate equal to that of other genotypes.

### Simulation experiment and statistical analysis

#### Experimental design

Simulations of the model described in Section Eco-evolutionary dynamics were carried out using discrete time intervals (one time step equalled to one day) and adding stochastic steps to account for possible drift when genotypes are at low frequencies. For each simulation, the pathogen population was initially composed of the wild host specialist which cannot infect the crop (*e*_*w*_ = 0.4 and *e*_*c*_ = 0). Epidemics were initiated by assuming that wild hosts were randomly infected with a probability of 0.01.

There were five input factors of interest (Table1): duration of the cropping season (5 values – 60, 120, 180, 240 and 300 days), the proportion (3 values – 2.5%, 5% and 10%), the aggregation level (2 values – random and aggregated) of the wild patches, the trade-off shape (3 values – 0.6, 1 and 1.4) and the mean dispersal distance of the pathogen (3 values – 2.5%, 10% and 25%). We set up a complete factorial design by considering each combination of the values of the five input factors. For each of these conditions, 20 replicates were simulated as follows: 5 landscape replicates by 4 model replicates. This led to a total of 5400 simulations. Note that more than 4 model replicates would be necessary for a refined study of the effects due to model stochasticity in a specific landscape structure. That was not the aim of this study, and we preferred to pool the model and landscape replicates to have sufficient degrees of freedom for the statistical analyses.

#### Outputs

Local dynamics were aggregated to give global evolutionary trajectories in the pathogen population (Fig.9). From these trajectories, we first characterized whether the emergence of a crop pathogen was successful through the variable *E* that equals 1 if a pathogen genotype that can infect the crop emerged and persisted in the simulation and 0 otherwise. Then, in the simulations where emergence occurred, we estimated the number of years that emergence required, *T*^*E*^, as the first year for which the proportion of healthy plants in the cultivated area dropped by 5%. Finally, we characterized mean aggressiveness of the crop pathogen population (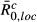) by averaging the aggressiveness 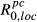 over the pathogen genotypes *p* in the pathogen population that developed on the cultivated host at equilibrium.

#### Statistical models

The effects of the input factors (cropping season duration, proportion and aggregation level of wild patches, trade-off shape and pathogen mean dispersal distance) on the descriptors of the pathogen evolutionary trajectory (*E*, *T*^*E*^, and 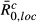) were assessed by fitting generalized linear models (GLM) with various link functions using the R software (R Core Team 2014) (Table2). As the number of successful emergences (*E* = 1) was very high within each combination of factors, it was not possible to estimate interactions for this variable. The GLMs used adequately fitted the data set as indicated by deviance residuals (Fig.10). In addition, they explained 77.2%, 97.6% and 99.4% of the deviance for *E*, *T*^*E*^, and 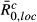, respectively.

**Table 2:** Summary of the generalized linear models used for the analysis of the three descriptors of the global pathogen evolutionary trajectories: *E*, emergence of a crop pathogen (*E*_*s*_ = 1 if emergence was successful in simulation *s* and 0 otherwise); *T*^*E*^, number of years required for the crop pathogen to emerge; 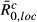, mean aggressiveness of the pathogen population on the crop at equilibrium

Descriptor	Distribution	Link function	Linear predictor
Emergence (*E*)	Binomial	Logit	−1 + LOC + PROP + BETA + CROP + DISP
Time before emergence (*T*^*E*^)	Gamma	Log	−1 + LOC + LOC:(PROP + BETA + CROP + DISP + PROP:BETA + PROP:CROP + PROP:DISP + BETA:CROP + BETA:DISP + CROP:DISP)
Pathogen aggressiveness on the crop ( 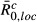 )	Normal	Natural	−1 + LOC + LOC:(PROP + BETA + CROP + DISP + PROP:BETA + PROP:CROP + PROP:DISP + BETA:CROP + BETA:DISP + CROP:DISP)

LOC, two-level factor of wild patches spatial aggregation (random or clustered); PROP, scaled wild host proportion; BETA, scaled parameter of the trade-off function (*β*); CROP, scaled cropping season duration; DISP, scaled pathogen mean dispersal distance; :, interactions.

## Results

### Observed outcomes

**Figure 5:**
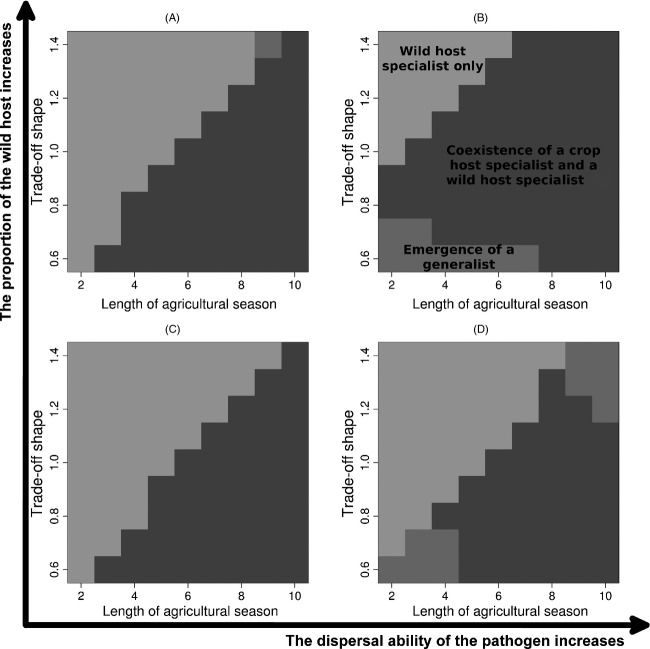
Patterns of pathogen diversity. The different plots represent different conditions in the proportion covered by the wild host and the pathogen dispersal ability: (A) high proportion of wild host (*q* = 10%) and low pathogen dispersal (*distM*^*P*^ = 2.5%); (B) high proportion of wild host (*q* = 10%) and high pathogen dispersal (*distM*^*P*^ = 25%); (C) low proportion of wild host (*q* = 2.5%) and low pathogen dispersal (*distM*^P^ = 2.5%); and (D) low proportion of wild host (*q* = 2.5%) and high pathogen dispersal (*distM*^*P*^ = 25%). These graphs represent the prediction of multinomial logistic regression models fitted to the simulated data set.

Over the entire simulation data set, four outcomes were observed: extinction of the pathogen population; persistence of the wild host specialist only; coexistence of one crop specialist and one wild host specialist; selection for one pathogen generalist (Fig.5). Extinction of the pathogen population only occurred in 1.8% of the simulations and corresponded to cases where pathogen dispersal ability was the highest and the proportion of wild hosts the lowest. These extinctions occurred at the very beginning of the simulations and were due to a small pathogen population size combined with demographic stochasticity. Selection for generalist pathogens was generally observed when the aggressiveness trade-off was weak and pathogen dispersal ability was at its highest values but was also possible with a strong trade-off when the cropping season was very long. These two contrasting situations led, respectively, to a generalist with low (in the first case) and high (in the latter case) aggressiveness on the crop. In all other contexts, pathogen evolution resulted in coexistence of a crop specialist and a wild host specialist when the agricultural season was long enough and the trade-off not too strong and to unsuccessful emergence of a crop pathogen otherwise.

### Effect of spatial configuration

The spatial configuration of the landscape was described by two of the five input factors: the aggregation level among wild patches and the proportion of area covered by the wild metapopulation. An increase in the aggregation of wild patches resulted in larger wild pathogen population sizes during the off season. Consequently, more new mutations were produced in more aggregated landscapes which facilitated the emergence of new pathogen genotypes able to thrive on the crop. Thus, more aggregated landscape patterns increased the number of successful emergences (Table3, intercept values) and decreased the time required for the crop pathogen to emerge (Table4, intercept values). However, selection during the off season was stronger and, over longer evolutionary time scales, increases in the aggregation level of wild host patches led to a small but significant lower mean aggressiveness of the pathogen population on the crop at equilibrium (Table4, intercept values).

**Figure 6:**
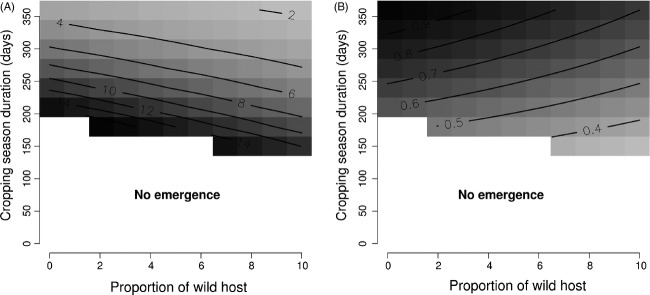
The effect of cropping season duration and the proportion of wild host in the agricultural landscape: (A) predicted values of the time (number of years) required to observe the emergence of a crop pathogen; and (B) relative mean aggressiveness of the pathogen population on the crop at equilibrium (0, the aggressiveness is minimal; 1 the aggressiveness is maximal). White: no successful emergence of a crop pathogen predicted by the model. Values for the grey scale are indicated by the contour lines.

Increases in the proportion of the landscape covered by patches of the wild host also increased pathogen population size because of increased wild host abundance. Thus, as for aggregation, this facilitated crop pathogen emergence (i.e. increased the number of successful emergences and decreased the time required for emergence to occur – Fig.6A and Tables3 and 4). At the same time, as its proportion increased, the wild host became an important habitat for the pathogen even during the agricultural season. As a consequence, increases in the proportion of the landscape covered by patches of the wild host decreased the mean aggressiveness of the pathogen population on the crop at equilibrium (Fig.6B and Table4). In addition, the effects of the proportion of the agricultural landscape covered by wild hosts on *T*^E^ and 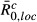 were more important when wild patches were randomly distributed in space (Table4). Indeed, as its spatial aggregation increased, the wild host metapopulation sustained a larger pathogen population which made the pathogen less sensitive to variations in wild host abundance.

### Effect of temporal heterogeneity

**Figure 7:**
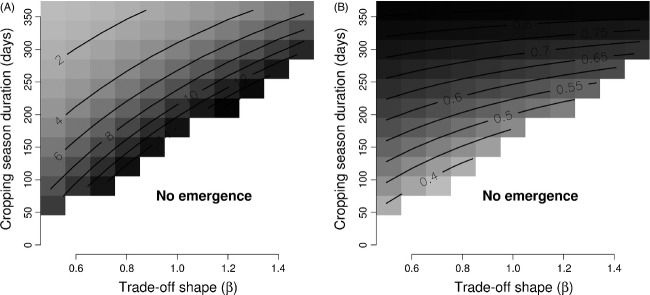
The effect of cropping season duration and trade-off shape: (A) predicted values of the time (number of years) required to observe the emergence of a crop pathogen, and (B) relative mean aggressiveness of the pathogen population on the crop at equilibrium (0, the aggressiveness is minimal; 1 the aggressiveness is maximal). Values for the grey scale are indicated by the contour lines.

The longer the cropping season, the greater the time period during which the cultivated host was available which decreased pathogen dependency on the wild host for survival. As a consequence, increased duration of the cropping season meant that more crop pathogen emergences were successful (Table3), the time required to observe crop pathogen emergence was shorter (Fig.7A and Table4) and the mean aggressiveness of the pathogen population on the crop at equilibrium was greater (Fig.7B and Table4). These effects on the pathogen evolutionary trajectory were similar regardless of whether the spatial distribution of wild host patches was random or aggregated (Table4). However, temporal heterogeneity mediated effects of the landscape spatial configuration (Table4). In fact, increasing the length of the cropping season resulted in both a decreased effect of the proportion of wild hosts in the landscape on *T*^*E*^ (time to emergence) and an increased influence of the proportion of wild hosts in the landscape on the mean aggressiveness of the crop pathogen population, 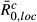.

### Effects of life-history traits

#### Trade-off in aggressiveness

Pathogen aggressiveness is a measure of pathogen fitness and reflects the potential disease severity a pathogen can cause to its hosts. The strength of the trade-off in aggressiveness on the two hosts (*β*) reflected the difficulty experienced by pathogen genotypes able to thrive on the crop with regard to survival during the off season on the wild host. Thus, increases in the strength of the trade-off (i.e. higher values of *β*) acted in the same way on the three variables describing the global pathogen evolutionary trajectory: a stronger trade-off reduced the chance (fewer emergences were successful) and increased the time of emergence (Fig.7A and Tables3 and 4) and decreased the mean aggressiveness of the pathogen population on the crop at equilibrium (Fig.7B and Table4). The effects of the trade-off in aggressiveness on *T*^*E*^ and 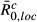 depended on the spatial configuration of the landscape and on the duration of the cropping season. Increases in the aggregation of wild patches, in the proportion of the agricultural landscape covered by wild hosts and in cropping season duration, resulted in a decreased impact of the trade-off shape on *T*^*E*^ and 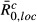 (Table4).

#### Dispersal

**Figure 8:**
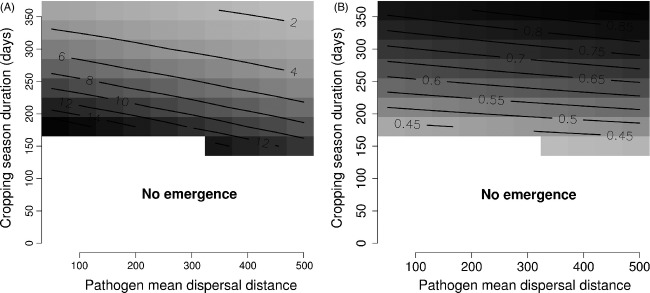
The effect of cropping season duration and pathogen mean dispersal distance: (A) predicted values of the time (number of years) required to observe the emergence of a crop pathogen, and (B) relative mean aggressiveness of the pathogen population on the crop at equilibrium (0, the aggressiveness is minimal; 1 the aggressiveness is maximal). White: no successful emergence of a crop pathogen predicted by the model. Values for the grey scale are indicated by the contour lines.

The larger the pathogen mean dispersal distance, the more pathogen propagules originating from a wild patch landed in crop fields. As a consequence, selection pressure posed by the crop increased with the pathogen's ability to disperse which facilitated the emergence of a crop pathogen (Table3), decreased the time required for crop pathogen emergence to occur (Fig.8A and Table4) and increased the mean aggressiveness of the pathogen population on the crop at equilibrium (Fig.8B and Table4). The spatial distribution of wild host patches did not change these effects on the pathogen evolutionary trajectory (Table4). Finally, increases in the scale of pathogen dispersal made the results more sensitive to changes in the proportion of the wild host in the landscape (Table4).

## Discussion

### Role of landscape spatial configuration

The interface between agricultural and natural elements of agro-ecological landscapes can vary tremendously in different systems (Fig.1), with the potential to significantly modify the way pathogens and their hosts interact. Modelling the spatial structure of the AE interface explicitly (described by the spatial aggregation of wild patches and the proportion of landscape they cover) enabled us to assess its effects on the emergence of a new crop pathogen and the potential for evolution towards increased aggressiveness (Fig.2). Interestingly, we found a trade-off between the managements of pathogen emergence and aggressiveness. Landscape structures that promoted larger pathogen populations on the wild host (high proportion and aggregation of remnant wild patches) facilitated the emergence of a crop pathogen by both increasing emergence event and decreasing emergence time. However, such landscape structures also decreased the selection pressure posed by the crop which resulted, over longer time scales, in reduced aggressiveness of the pathogen population on the crop at equilibrium. Note that these results hold for a relatively low proportion (<10%) of wild hosts in the landscape. For higher proportion and lower spatial aggregation, remnant wild patches may hamper disease spread at the landscape scale by decreasing the level of connectivity among crop fields (Papaïx et al. 2014c). More generally, wild patches can act as stepping stones or refugia for parasitoids and predators of vectors and pests to provide better biological control at the landscape scale (Woltz et al. 2012; MacFadyen and Müller 2013).

Trade-offs between the ecosystem services provided by remnant wild patches in agro-ecosystems are generally the rule as ecosystem services are not independent from each other (Millennium Ecosystem Assessment 2005). For example, Alexander et al. (2014) noted in the context of plant viruses that, while reduction in the abundance of both weeds and wild host plants can be beneficial for controlling viral diseases in crops, large-scale removal of noncrop plants also reduces heterogeneity in agro-ecological landscapes. This can influence the evolution and spread of viruses and thus potentially has negative as well as positive management consequences. As pointed out by Power (2010), these trade-offs should be considered in terms of spatial and temporal scales for management purposes. The spatial scale of emergence is generally local but depends on the spatial configuration of wild patches. Indeed, an aggregated distribution of local patches within the wild metapopulation could lead to more localized emergences than when patches are randomly distributed as the AE interface is more concentrated. In addition, emergence is a short-term evolutionary process. Once the pathogen has successfully shifted onto the crop, further mutations towards more aggressive pathotypes are more difficult to manage as they occur at larger spatial and longer temporal scales. Thus, with respect to the example we provide here, increasing the aggregation of wild vegetation within agricultural landscapes may be a useful approach to hinder pathogen evolution towards aggressive pathotypes at larger spatial and longer time scales while additional management strategies may be needed at local spatial scales and over shorter timeframes to control emergence (e.g. monitoring of pathogen populations).

### Role of temporal heterogeneity

As expected, we found that longer agricultural seasons facilitated the emergence of a crop pathogen and increased its level of adaptation on the crop. Indeed, the perennial wild host enables the pathogen to survive during the off season for the crop. This situation generates a tension between the advantage (to the pathogen) of increased ability to attack crop hosts, but the potential disadvantage (when there are trade-offs) of a reduced ability to persist on wild hosts (which can represent an important refuge in the off season). Obviously, as the time period during which the crop was available increased, the pathogen's dependency on the wild host for survival decreased, which resulted in a better adaptation of the pathogen to the crop. Life history is, however, critical here: if a pathogen is less constrained (e.g. no trade-off or the pathogen can survive off-host as a saprophyte) then presumably generality (in host range) will be more likely to emerge. Consistent with this, van den Berg et al. (2010, 2011) showed that a longer period of host absence selected for higher transmission rates in the presence of a trade-off between transmission and virulence but lower transmission rates in the presence of a trade-off between transmission and off-season survival. The role of the duration of the cropping season has direct consequences for the management of agricultural landscapes. For example, an increase in crop presence using crop varieties sown during the fall in temperate agricultural systems could increase the risk of disease emergence and the global adaptation of pathogen populations to crop hosts. More generally, crop rotations over time, that is inter- or intraseasonal changes in the crops, are known to impact disease dynamics in the long term and are recommended to provide disease breaks (Bennett et al. 2012). However, the efficiency of such rotations could be limited in regions where the crop is cultivated over large areas and for an aerial initial inoculum.

A key question concerning the off season for the crop that was not addressed here is how homogeneous the vegetation gap is across the landscape (Alexander et al. 2014). Indeed, different crops or crop cultivars can be planted and harvested at different times leading to a mosaic of host presence. In addition, self-sown volunteers and remnant plants can help obligate biotroph pathogens to bridge the gap when most crops are harvested. For example, volunteer wheat plants heavily infected with leaf rust (*Puccinia triticina*) are commonly observed (Burleigh et al. 1969; Mehta and Zadoks 1970; Moschini and Pérez 1999; Singh et al. 2004) indicating that the pathogen can survive locally and bridge the off season, resulting in the early appearance of rust infection in newly planted fields (Eversmeyer and Kramer 2000; Goyeau et al. 2006). Eversmeyer and Kramer (2000) suggested that the destruction of volunteer wheat would significantly reduce primary inoculum sources and disease severity as severe damage in wheat fields is observed when scattered plants are left when the new crop is planted. However, no information is available on the role of such volunteer plants in determining the year-to-year genetic structure of pathogen populations, and, in particular, in accelerating or hampering the fixation of new mutations or the rate of resistance breakdown.

In the same way, the demographic and genetic processes acting during the off season in the remnant wild vegetation patches of agro-ecological systems have received almost no consideration and we thus have little understanding of the role of the off season in shaping disease dynamics and pathogen genetic structure. Abiotic conditions during the off season are known to be important in determining pathogen survival and in-season epidemics (Marçais et al. 1996; Penczykowski et al. 2015). Biotic conditions can also play a critical role as exemplified by the case of barberry and stem rust discussed in the Introduction (Roelfs 1982). In addition, different pathogen genotypes may vary in their response to the off-season environment due to differential selection between pathogen life-history stages (Sommerhalder et al. 2011). This led Tack and Laine (2014) to extend the classic disease triangle to the off season. They developed their view with a meticulous investigation of the off-season dynamics of powdery mildew *Podophaera plantaginis* of *Plantago lanceolata* in which they found that pathogen survival during that time was affected by both environmental and spatial factors. Their results also suggested the presence of local adaptation by the pathogen to its local off-season environment. This opens the way for a better understanding and integration of off-season mechanisms in pathogen evolution and disease dynamics.

### Role of life histories

Plant pathogens exhibit a diverse array of dispersal mechanisms which suggests that different host–pathogen interactions may occur over a broad range of spatial scales (Thrall and Burdon 1997). This aspect of pathogen life history thus has the potential to strongly mediate the effects of spatial structure on both disease epidemiology and pathogen evolutionary trajectories [e.g. Thrall and Burdon (1999, 2002)]. We found here that increases in the mean dispersal distance of the pathogen facilitated its shift from the wild host to the crop as well as its evolution on the crop towards more aggressive pathogen genotypes. In addition, increases in the scale of pathogen dispersal made the results more sensitive to changes in the proportion of the wild host in the landscape which is consistent with the literature (Papaïx et al. 2013). These results can be explained by the fact that greater pathogen dispersal distances increase the rate of propagule exchange between cropping and wild elements of agricultural systems. Indeed, the first pathogen genotypes arriving on the crop from the wild host can cause mild symptoms on the crop but cannot develop their own epidemic because their growth rate on the crop is negative (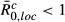). Spore dispersal generates source–sink dynamics between wild and crop hosts. As pathogen dispersal distance increases, a larger pathogen population can be maintained on the crop due to increased spillover from the wild host (Holt 1993) which accelerates the evolution and the emergence of pathogen genotypes specialized on the crop.

Equally important are trade-offs between different life-history traits or in the ability to infect different hosts. The role of such a trade-offs has been studied using an adaptive dynamics theory approach in a nonspatial context (van den Berg et al. 2010, 2011; Hamelin et al. 2011). van den Berg et al. (2010, 2011) did not found situations in which coexistence of different pathogen genotypes was possible. Conversely, Hamelin et al. (2011) found that evolutionary branching in the pathogen population was possible due to the appearance of negative density dependence in the season-to-season dynamics but required that the trade-off between transmission and off-season survival has a concave shape (weak trade-off). Consistent with this, we found that when the trade-off between the adaptation to the wild host and the crop was weak, emergence and subsequent coexistence of two pathogen genotypes was the rule over a range of values for the length of the cropping season. Nevertheless, under a strong trade-off (convex shape), coexistence between a crop specialist and a wild host specialist was also observed but required a longer cropping season. This difference was probably due to the explicit consideration of the population spatial structure that favours the maintenance of diversity by means of several mechanisms (Sasaki et al. 2002; Salathé et al. 2005; Abrams 2006; Brown and Tellier 2011; Débarre and Lenormand 2011; Tellier and Brown 2011; Zhan and McDonald 2013). In addition, we have also shown that the spatio-temporal pattern of the landscape influences the speed of pathogen evolution and the level of adaptation of the pathogen population at equilibrium.

The analysis we reported here focused on the role of pathogen dispersal ability and on the existence of possible trade-offs in the ability to infect different hosts. Obviously, predictions for how crop composition and landscape structure affect pathogen interactions with (and reliance on) wild and cultivated hosts are also dependent on life-history traits other than dispersal and trade-offs, including for example mating system, transmission mode and the presence of saprophytic stages – all have the potential to modify pathogen persistence, population size and/or rates of evolution. For example, the ability of the wild host to disperse was fixed but the spatial scale of dispersal of both the host and the pathogen directly influence disease dynamics and plant pathogen co-evolution in the wild metapopulation. Low host and pathogen dispersal abilities imply a high level of asynchrony in disease dynamics among local populations with frequent local extinction and recolonization events and can result in a greater host and pathogen diversity (Thrall and Burdon 2002; Papaïx et al. 2014b). Conversely, as the host (respectively, the pathogen) dispersal ability increases severe boom and bust dynamics dominate due to a high level of synchrony among local populations, resulting in maladaptation of the pathogen (respectively, the host) population (Gandon 2002; Thrall and Burdon 2002; Papaïx et al. 2014b). Such different patterns of disease and host–pathogen co-evolutionary dynamics in wild patches are likely to have consequences for further pathogen evolution on the crop and need specific consideration to better predict the emergence of new crop pathogens.

### Limitations: consideration of genetic diversity in host communities

As a first approximation, in this study, we considered a genetically homogeneous crop. The use of crop diversity through spatial diversification schemes and rotations is an active field of research focused on reducing disease severity and the evolutionary potential of pathogen populations (Zhan et al. 2014). From a management perspective, the spatio-temporal structure of crop diversity could be used in two different ways. First, crop diversity directly influences connectivity among cropping components and can delay the spread of the pathogen population (Skelsey et al. 2010; Papaïx et al. 2014c). This can result in a smaller pathogen population size on the crop with a lower survival probability during the off season (Suffert et al. 2011) and a lower evolutionary potential (Zhan et al. 2014). Second, crop diversity could directly influence the level of adaptation of the pathogen population to different crop cultivars and its genetic diversity (Marshall et al. 2009; Papaïx et al. 2011), potentially impacting its interaction with the wild host.

Heterogeneity within remnant wild vegetation should also be considered as wild plant communities are frequently very complex, being composed of native species, crop relatives and weedy exotics that can impose different selection pressures on pathogen populations (Mitchell and Power 2006; Thrall et al. 2007; Moore and Borer 2012; Seabloom et al. 2013). In addition, wild plant community dynamics differ from crops significantly in terms of population size, density and spatial distribution, genetic variability, and population continuity or predictability through time (Burdon 1993). Environmental conditions are also less stable potentially resulting in drastic fluctuations in population sizes and suboptimal growth conditions at least some of the time. All together these generally result in a marked stochasticity of pathogen dynamics at a local scale with repeated extinction/recolonization in individual demes even if disease dynamics appear stable at broader spatial scales. From an evolutionary perspective, the wild part of the AE interface is far from a homogeneous landscape (Burdon and Thrall 2014) and is better characterized as a mosaic of selection forces and intensity with different demes representing coevolutionary hot and cold spots (Thompson 1999; Smith et al. 2011). The spatial scale of local adaptation of both pathogen and plant populations can also be highly variable (Laine 2005; Jousimo et al. 2014) depending on other life-history attributes. Hence, it would be interesting to extend the present approach to consider more diverse situations for the wild elements (e.g. two wild hosts with some heterogeneity among host patches).

## Figures and Tables

**Figure A1:**
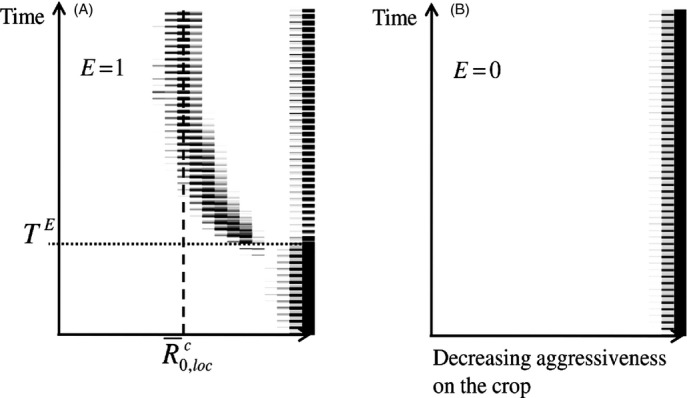
Examples of global evolutionary trajectories in the pathogen population when the emergence of a crop pathogen is successful (A, *E* = 1) or not (B, *E* = 0). In A, the time that the emergence required (*T*^*E*^) and the mean aggressiveness of the stable pathogen population on the crop (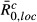) are also displayed. The grey intensity indicates the frequency of pathogen genotypes (*x*-axis) across time (*y*-axis), white: the frequency is equal to 0, black: the frequency is equal to 1.

**Figure A2:**
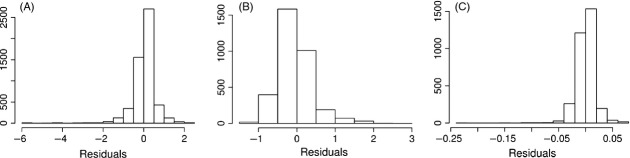
Histograms of deviance residuals for *E* (A), *T*^*E*^ (B) and 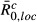 (C). The models are specified in the Table2 of the main text.

## Appendix A Statistical results

**Table A1:** Estimated effects of the proportion and the aggregation level of the wild patches, the trade-off shape, the cropping season duration and the pathogen mean dispersal distance on the emergence of a crop pathogen (*E* – Table2 of the main text)

Effect	Mean (Confidence interval at 95%)
Intercept	
Random	1.76 (1.56,1.97)
Clustered	2.61 (2.37,2.86)
Wild host proportion (*q*)	0.96 (0.83,1.10)
Trade-off shape (*β*)	−4.91 (−5.27, −4.56)
Cropping season duration (*Y*)	5.25 (4.89,5.63)
Pathogen mean dispersal distance (*distM*^*P*^)	1.09 (0.95,1.24)

**Table A2:** Estimated effects of the proportion and the aggregation level of the wild patches, the trade-off shape, the cropping season duration, the pathogen mean dispersal distance and interactions on the time required for the emergence of a crop pathogen (*T*^*E*^) and the mean aggressiveness of the stable pathogen population on the crop ( – Table2 of the main text)

Effect	Mean (Confidence interval at 95%)			
	Time before emergence (log scale)		Mean aggressiveness (^*^10^−1^)	
	Random	Clustered	Random	Clustered
Intercept	1.78 (1.75,1.82)	1.67 (1.64,1.70)	2.20 (2.19,2.21)	2.13 (2.12,2.14)
Wild host proportion (*q*)	−0.24 (−0.26, −0.21)	−0.17 (−0.19, −0.14)	−0.25 (−0.26, −0.24)	−0.21 (−0.22, −0.20)
Trade-off shape (*β*)	0.62 (0.58,0.65)	0.55 (0.52,0.59)	−0.21 (−0.22, −0.20)	−0.17 (−0.18, −0.16)
Cropping season duration (*Y*)	−0.66 (−0.69, −0.62)	−0.68 (−0.71, −0.65)	0.59 (0.58,0.60)	0.58 (0.57,0.59)
Pathogen mean dispersal distance (*distM*^*P*^)	−0.25 (−0.28, −0.23)	−0.24 (−0.27, −0.22)	0.06 (0.05,0.07)	0.05 (0.04,0.06)
*q* × *β*	−0.06 (−0.09, −0.04)	−0.06 (−0.09, −0.04)	0.04 (0.03,0.05)	0.02 (0.01,0.03)
*q* × *Y*	0.03 (0.01,0.06)	0.04 (0.01,0.07)	−0.08 (−0.09, −0.07)	−0.07 (−0.08, −0.06)
*q* × *distM*^*P*^	−0.04 (−0.07, −0.01)	−0.05 (−0.08, −0.03)	−0.03 (−0.04, −0.03)	−0.01 (−0.02,0.00)
*β* × *Y*	−0.10 (−0.14, −0.07)	−0.11 (−0.15, −0.08)	0.11 (0.10,0.12)	0.10 (0.09,0.11)
*β* × *distM*^*P*^	−0.13 (−0.16, −0.10)	−0.12 (−0.15, −0.09)	0.06 (0.05,0.07)	0.06 (0.05,0.07)
*Y* × *distM*^*P*^	0.03 (0.00,0.05)	0.06 (0.03,0.08)	0.03 (0.03,0.04)	0.01 (0.00,0.02)

×, denotes interactions.
